# Factors Leading Municipal Authorities to Implement Preventive Interventions for Lyme Disease

**DOI:** 10.3390/ijerph16091547

**Published:** 2019-05-01

**Authors:** Johann Jacob, Pierre Valois, Cécile Aenishaenslin, Catherine Bouchard, Sandie Briand, Denis Talbot, Maxime Tessier

**Affiliations:** 1 Observatoire Québécois de L’adaptation aux Changements Climatiques (OQACC), Faculté des Sciences de L’éducation, Université Laval, Québec, QC GIV 0A6, Canada; johann.jacob@fse.ulaval.ca (J.J.); maxime.tessier@fse.ulaval.ca (M.T.); 2 Groupe de Recherche en épidémiologie des Zoonoses et Santé Publique (GREZOSP), Faculté de Médecine Vétérinaire (FMV), Université de Montréal, Saint-Hyacinthe, QC J2S 2M1, Canada; cecile.aenishaenslin@umontreal.ca (C.A.); catherine.bouchard@canada.ca (C.B.); 3 Public Health Risk Sciences Division, National Microbiology Laboratory, Public Health Agency of Canada, Saint-Hyacinthe, QC J2S 2M1, Canada; 4 National Public Health Institute of Quebec (INSPQ), Montréal, QC H2P 1E2, Canada; sandie.briand@inspq.qc.ca; 5 Département de Médecine Sociale et Préventive, Faculté de Médecine, Université Laval, Québec, QC G1V 0A6, Canada; denis.talbot@fmed.ulaval.ca; 6 Unité Santé des Populations et Pratiques Optimales en Santé, Centre de Recherche du CHU de Québec – Université Laval, Québec, QC G1S 4L8, Canada

**Keywords:** municipal, climate change, Lyme disease, attitude, theory of planned behavior

## Abstract

The aim of this study is to document climate change adaptation interventions targeting Lyme disease at the municipal level in the province of Quebec (Canada). This exploratory study relies on the theory of planned behavior and certain constructs from the health belief model to identify the factors leading municipal authorities to implement preventive interventions for Lyme disease (PILD). Data were obtained from an online survey sent, during the summer of 2018, to municipal officers in 820 municipalities in Quebec, in all health regions where the population is at risk of contracting Lyme disease (response rate = 36%). The questionnaire was used to measure the implementation of PILD, the intention to implement these interventions, attitudes, perceived social pressure, perceived control (levers and barriers) over interventions, perceived effectiveness of preventive measures, risk, and perceived vulnerability. Results of structural equation analyses showed that attitudes were significantly associated with municipal authorities’ intention to implement PILD, while the intention to implement PILD was a significant predictor of the implementation of PILD. Additional analyses showed that perceived barriers added a moderating effect in the intention-implementation relationship. The prediction of behaviors or practices that municipal authorities could implement to prevent Lyme disease will enable the evaluation over time of the evolution of Quebec municipalities’ adaptation to Lyme disease. Moreover, the examination of the associations of specific psychosocial factors revealed important implications for the design of effective behavior-change interventions, which would allow health officials doing awareness work to create personalized interventions better suited to municipal officers and their specific contexts.

## 1. Introduction

Beyond extreme weather events, such as heat waves, droughts, floods, and storm surges, climate change is also linked to an increased prevalence of vector-borne diseases, such as Lyme disease [1,2,3]. *Ixodes scapularis* ticks infected with *Borrelia burgdorferi* can transmit it to species, including humans, which can develop Lyme disease. Symptoms of the disease can include the appearance of *erythema migrans* (redness on the skin at the site of the tick bite; Ogden et al., 2009 [4]), which may be accompanied by fever, fatigue, headache, stiffness in the neck, muscle aches, and joint pain. Without early treatment, the disease can cause systemic problems, such as arthritic, cardiac, and neurological disorders [5]. 

Lyme disease, the most commonly reported vector-borne disease in temperate regions, is emerging in Canada [6,7,8]. Since the disease was first identified in the province of Quebec (Canada) in 2007–2008 [9,10], the number of reported human cases has been increasing. This increase is also strongly associated with the expansion of *Ixodes scapularis* populations [6]. In 2011, the proportion of reported cases of Lyme disease acquired locally was 16% (5/32), compared with 73% (219/301) in 2018 [11]. 

Adaptation refers to “the process of adjustment to actual or expected climate and its effects, in order to moderate harm or exploit beneficial opportunities” [12]. Health-related adaptation involves any intervention (for example developing spatial modeling of geo-referenced climate and environmental information) aiming to reduce the health burden associated with climate change [13]. Given the emergence and major impacts of Lyme disease on population health, there is a need to implement structures aimed at fostering the populations’ adaptation to this problem. 

Despite the importance of climate-related health risks, there are few studies, including systematic examinations of the current state of health-related adaptation in Canada [14,15,16]. Although the role of local authorities and stakeholders, such as municipalities, in climate change adaptation has been emphasized [17,18,19,20,21,22], a large part of the literature on climate change adaptation at the municipal level tends to focus on what “should be done” and not on what “is actually done” [23]. However, to control and prevent vector-borne diseases, developing adequate response plans, enhancing surveillance systems, and developing effective and locally appropriate strategies are essential [3].

Municipalities in Quebec do not have a mandate to protect public health. Surveillance, prevention, and control of infectious diseases are provincial responsibilities, under the supervision of the Ministry of health and social services (*Ministère de la Santé et des Services sociaux*). These responsibilities are implemented in collaboration with health and social services regional centers (*Centres de santé et de services sociaux*) that have their own public health departments (PHD; *directions de santé publique*). Regarding Lyme disease, regional PHD inform municipalities about risks and share communication tools. Regional PHD can also provide certain resources to municipalities. For instance, health officers can go to municipalities to train and inform resource persons. Municipalities are therefore encouraged to act through regional PHD but have no official mandate at this time. That said, some municipalities are proactive regarding Lyme disease, through climate change adaptation plans that they develop, by integrating public health issues into their priorities. For example, the City of Montreal has announced its intention to protect its workers from Lyme disease. In addition, to our knowledge, at least one municipality has decided to finance, within its own budget, an integrated project to reduce the risk of Lyme disease.

Some empirical work has been conducted to document climate change adaptation interventions at the level of municipalities or civil society organizations in Canada (to our knowledge, none specifically targeting vector-borne disease interventions), but from secondary data, such as official documents, including adaptation plans, contingency plans, and information on official websites [16,24]. Other empirical work has also been limited to a few major Canadian cities [16,25]. There was thus a need to undertake a study among municipal staff (such as heads of land use planning and environmental advisors) of all Quebec municipalities where the population is exposed to Lyme disease. The purpose of this exploratory study is to document climate change adaptation interventions targeting Lyme disease at the municipal level in the province of Quebec (Canada).

### 1.1. Preventive Interventions for Lyme Disease (PILD)

In the absence of an available vaccine, preventive interventions for Lyme disease (PILD) can be divided into two categories: Preventive environmental measures aimed at reducing the density of infected ticks in the environment [26,27,28] and measures promoting the adoption of preventive behaviors by individuals through awareness-raising in at-risk populations [27,29]. Preventive environmental measures include, among others, direct actions on tick populations (e.g., use of acaricides), landscaping actions, actions targeting wild animal species that are reservoirs of the agents or reproduction hosts for the ticks (e.g., reduction in the density of deer populations, exclusion of deer from inhabited areas, treatment of deer and small rodents against ticks, vaccination of rodents against the *Borrelia* bacteria).

Lyme disease preventive behaviors are often unevenly adopted by residents of high-risk areas [30], including Canada [31,32]. For this reason, public health organizations are conducting education campaigns aimed at promoting the adoption of preventive behaviors through awareness-raising. For instance, in the province of Quebec, public health agencies make information on Lyme disease available to municipalities for dissemination through their website to their population. This information includes a description of ticks, the disease, and its symptoms; possible protection and prevention measures; tips for removing the tick, etc.

Whereas evaluations of existing response strategies in public health systems are scarce [33], potential factors constraining or facilitating the planning or implementation of these interventions over time and across localities remain to be investigated [34]. From a health education perspective, a better understanding of the determinants that favor or limit the implementation of preventive interventions by local decision makers would facilitate the identification of factors that government authorities should consider when designing information tools aimed at changing municipal actors’ knowledge, attitudes, and beliefs [34,35,36]. 

### 1.2. Theoretical Models Explaining the Implementation of PILD

Behavioral change invariably requires an understanding of the factors that motivate these changes [37]. Empirical work based on psychosocial models predicting behaviors, such as the theory of planned behavior, that is, TPB [38], and the health belief model, HBM for short [39,40], has made its way into the climate change adaptation literature to help researchers understand what drives action [41,42]. However, the use of such models in research linked to Lyme disease is still rare [26,29,31,34]. From an awareness and intervention perspective, such studies are essential for identifying malleable factors that offer a lever for change, in order to better target interventions.

Among the various theoretical models, the HBM [39,40] is one of the most used to study the influence of factors explaining the adoption of behaviors to prevent diseases and health problems. As for the TPB [38], its usefulness for predicting behaviors in various contexts has been repeatedly demonstrated [43,44]. The same is true regarding its usefulness for better understanding of pro-environmental behaviors, that is, behaviors that inhibit or foster sustainable, climate-healthy, and nature-enhancing choices [45].

The links between knowledge of risk, perceived risk, attitudes, and adoption of PILD (at the individual or household level) have already been studied in the literature [28,29,46,47,48]. Several studies have also included the examination, in individuals, of the determinants of social acceptability of certain measures related to Lyme disease or other vector-borne diseases [26,49,50]. However, to our knowledge, this type of study has never been conducted at the municipal level, nor has any study modeled all the psychological and organizational processes leading to the adoption of PILD. 

### 1.3. TPB Constructs

**Figure 1:**
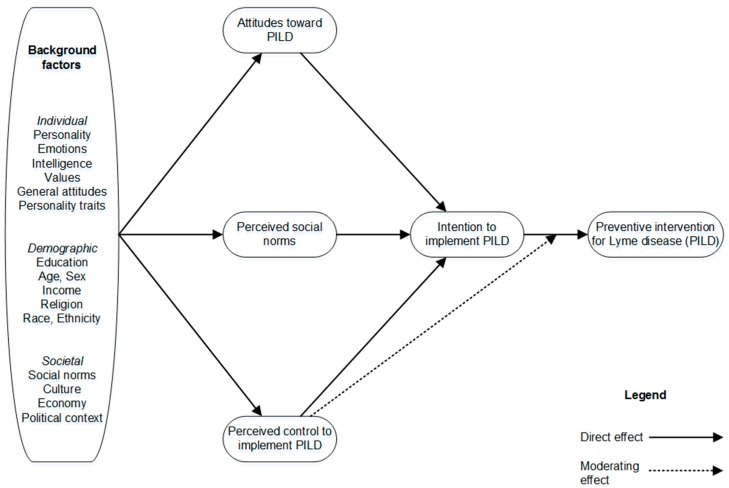
The theory of planned behavior model.

According to the TPB (see Figure 1), intention to implement PILD and perceived behavioral control (i.e., municipal officers’ perceptions of the degree to which their municipality is capable of, or has control over, implementing a given intervention) are the immediate antecedents (i.e., precursors) of these interventions. 

Theoretically, perceived behavioral control—to the extent that it accurately reflects actual control—is, like actual control, expected to moderate the effect of intention on behavior or action [51]. In other words, actual control over the action could affect the strength of the relation between the intention and action. Indeed, according to Fishbein and Ajzen [52], the effect of intention on action should be stronger when actual control is high rather than low. For instance, when municipal officers believe that their municipality actually has control over the implementation of PILD, they tend to act in accordance with their intentions. Inversely, if municipal officers believe that they have low control over the implementation of PILD, they do not tend to act in accordance with their intentions [53]. In Figure 1, this moderating effect is illustrated by a dash arrow between the variable called “perceived behavioral control to implement PILD” and the direct effect (solid arrow) of the “intention to adopt PILD” variable on the implementation of PILD.

The TPB also postulates that municipal authorities’ intention to implement PILD should increase to the extent that they hold favorable attitudes toward such interventions, think that significant others (e.g., the municipality’s population; regional, provincial, or federal public health agencies) support these interventions (i.e., perceived social norms), and perceive that the municipality has control over them.

### 1.4. Background Factors: Perceived Severity and Vulnerability

The TPB also proposes that a multitude of background factors (e.g., ethnicity, socioeconomic status, education, personality) can potentially influence people’s beliefs (Figure 1). Thus, to gain further insight into the underlying reasons for the implementation of PILD among municipal officers, we examined the potential impact of two background factors from the health belief model: The perceived risk or vulnerability represented by the disease (perceived by municipal officers), the perceived impact of the disease (severity) on health (again perceived by municipal officers) [39,40].

### 1.5. The Present Study

The purpose of this article is therefore to examine the factors leading municipal authorities to implement PILD. Accordingly, the following hypotheses were developed:

(1) Intention to implement PILD is the immediate antecedent of these interventions.
(2) Municipal officers’ intention to implement PILD should increase to the extent that they hold favorable attitudes toward such interventions, think that significant others support these interventions (i.e., perceived social norm), and perceive that the municipality has control over them.
(3) Valois et al. [54] showed that Quebec’s municipal officers have little control over the adoption of adaptive interventions for heat and flooding. Thus, we expect the relationship between municipal officers’ intention to implement PILD and their actual implementation of PILD to be moderated by the perceived barriers and perceived behavioral control (i.e., municipal officers’ perceptions of the degree to which their municipality is capable of, or have control over, implementing a given intervention). More specifically, we formulated the hypothesis that when municipal officers believe that their municipality actually has control over the implementation of PILD, they will tend to act in accordance with their intentions.
(4) A high level of perceived risk (vulnerability) represented by Lyme disease and a high level of perceived impacts of Lyme disease (severity) on health is expected to be positively related to intentions to implement PILD. The effects are expected to be indirect, operating via attitude toward PILD, perceived social norms regarding PILD, and perceived control to implement PILD.

## 2. Materials and Methods 

### 2.1. Participants

The target population in this exploratory study consisted of the 820 municipalities in the province of Quebec (Canada) where there is a higher risk for Lyme disease associated with the presence of established *I. scapularis* vector tick population (illustrated by the presence of at least three human cases acquired locally and reported to public health authorities, or 23 people with tick bites in the last five years, or a demonstration of tick population establishment through active vector surveillance), according to the provincial map of risk of Lyme disease acquisition [55]. This list also included the 19 Montreal boroughs. We limited ourselves to these municipalities because municipal authorities were more likely to feel concerned by the study, given the higher prevalence of Lyme disease. This study adopts a cross-level design, as we are assessing the relationship between independent and dependent variables at two different levels (individual and organizational) [56,57,58]. Hence, the responses of individual municipal officers were used as a proxy for municipalities’ level of PILD [59]. In the summer and fall of 2018, we sent the municipal officers an invitation to complete an online questionnaire (LimeSurvey). The first person contacted was asked to transfer the survey if he or she felt that someone else in the organization would be more able to respond. It is difficult to identify the right person to contact and collect information to document climate change interventions targeting Lyme disease at the municipal level in the province of Quebec. For instance, some municipalities do not have an environmental advisor. There is also a high turnover rate among municipal staff. Although our method of contacting municipalities is not free of bias, it was the only strategy that would result in sufficient data to build an index. Hence the exploratory nature of our study. We are, however, confident in the portrait generated. 

A total of 293 respondents (93 women, 31.74%; 127 men, 43.34%; 73 declined to give their gender, 24.91%) were surveyed between July 12 and October 12, 2018. The response rate was 36%. Table 1 presents the response rates by the Quebec health region. It is worth mentioning that in 2018, most cases of Lyme disease (reported and acquired in Quebec) were likely acquired in Montérégie (93/219; 42%), Estrie (87/219; 40%), and Mauricie-Centre du Québec (17/219; 8%) [11].

Table 2 presents the breakdown of sample sizes by municipality size. The respondents were working mostly for small and medium-size municipalities. A proportion of 6% of the sample included respondents from municipalities with a population above 50,000 inhabitants (n = 18). This proportion is similar to that found in the health regions under study. As for the occupation, the respondents were responsible mostly for development and land-use planning (30.38%), urbanism (43.34%), the environment (39.93%), or sustainable development (23.21%). More than one answer was possible for this question. The mean number of employees in the department where the respondents worked was 19 (SD = 91.1). For 46.42% of the participants, the highest educational level obtained was a university degree; 9.22% reported being under 30 years old, 17.06% between 30 and 39 years old, 23.55% between 40 and 49 years old, 19.45% between 50 and 59 years old, and 11.26% 60 years old or over. Most respondents had been working in the municipal sector, as well as in their current municipality, for three to 10 years.

### 2.2. Questionnaire

The questionnaire administered to the municipal officers assessed the TPB constructs, as well as some HBM constructs through the perceptions of municipal officers who answered the survey.

#### 2.2.1. Proximal Determinants of Intention and Implementation of PILD

*Attitude toward the implementation of PILD.* Responses to three questions were used as reflective indicators of municipal officers’ attitudes toward the implementation of PILD (reliability Cronbach alpha = 0.74). The reliability Cronbach alpha provides an estimate (varying between 0 and 1) of the homogeneity (internal consistency) of a measurement instrument or a latent variable composed of a set of items that all putatively measure the same construct or concept. A Cronbach alpha value close to 1 indicates a high level of internal consistency among the items measuring the construct. 

“In general, the majority of elected officials in our municipality believe that”: (ATT-1) municipalities have an important role to play in protecting the public against Lyme disease, (ATT-2) protecting the population against Lyme disease should be among our municipality’s priorities, and (ATT-3) our municipality should take action to reduce the density of infected ticks in the areas under its responsibility. Participants rated each item on a four-point scale ranging from “strongly disagree” (+1) to “strongly agree” (+4).

*Perceived social norms regarding PILD.* Responses to three questions were used to assess municipal officers’ perception of social norms related to the implementation of PILD (reliability Cronbach alpha = 0.90): “Using the following response scale, please indicate how much you agree or disagree with the following statements”: (N-1) Regional bodies to which the municipality belongs have high demands regarding our municipality’s actions to protect the population against Lyme disease, (N-2) Regional, provincial, and federal public health authorities have high demands regarding our municipality’s actions to protect the public against Lyme disease, and (N-3) The population of our municipality has high expectations regarding our action to protect the population against Lyme disease. Participants rated each item on a four-point scale ranging from “strongly disagree” (+1) to “strongly agree” (+4).

*Perceived control over the implementation of PILD*. The questionnaire addressed seven control factors. Participants rated each item on a four-point scale ranging from “strongly disagree” (+1) to “strongly agree” (+4) (reliability Cronbach alpha = 0.80): “Using the following response scale, please indicate how much you agree or disagree with the following statements”: (B-1) The role of municipalities in protecting the public against Lyme disease is unclear, (B-2) there is no consensus among specialists on the methods and techniques to be put in place to protect the population against Lyme disease, (B-3) there is little concrete and useful information on the protection of the population against Lyme disease in the official documentation, (B-4) employees in our municipality are too overburdened with their basic responsibilities to spend time protecting the public against Lyme disease, (B-5) actions to protect the population against Lyme disease are in areas beyond the scope of the municipalities, (B-6) it is difficult to achieve joint action with the other actors in our region in terms of protecting the population against Lyme disease, (B-7) it is difficult to have an internal budget to fund measures to protect the population against Lyme disease.

#### 2.2.2. Background Factors

Background factors refer, for instance, to demographics, personality, beliefs, and other individual differences that can influence behavior indirectly by influencing a person’s behavioral and normative beliefs. In our model, perceived vulnerability and perceived severity, as perceived by municipal officers, were included as background factors.

*Perceived vulnerability.* First, the respondents were asked the following question: “Do you believe it is possible to contract Lyme disease in your municipality?” For those who answered “Yes,” a second question assessed their perceived risk severity on a five-point scale ranging from “very low” (+1) to “very high” (+5). A score of 0 was given to the respondents who answered “No” to the first question.

*Perceived severity.* Responses to two questions were used as reflective indicators of perceived severity (correlation coefficient r = 0.81): (S-1) “If a person has Lyme disease, do you think the negative impact on his/her physical health would be…,” (S-2) “Do you think that the negative consequences on that person’s mental health would be…” Participants rated each item on a four-point scale ranging from “No negative consequences” (+1) to “very severe consequences” (+4).

#### 2.2.3. Dependent Variables

*Intentions.* Intentions were assessed by computing the mean response to the following three items (reliability Cronbach alpha = 0.94): “Using the following response scale, please indicate how much you agree or disagree with the following statements. As part of my duties:” (INT-1) “I intend to prioritize the protection of the population against Lyme disease over the next three years,” (INT-2) “I am determined to take action to prioritize the protection of the population against Lyme disease over the next three years,” and (INT-3) “I plan to regularly propose measures to protect the population against Lyme disease over the next three years.” Responses were provided on four-point scales ranging from “strongly disagree” (+1) to “strongly agree” (+4).

*PILD Index.* To measure the PILD adopted by municipal authorities, we developed and validated an index, hereinafter named PILD index. It is formed from a set of indicators referring to interventions that municipal authorities could adopt to prevent Lyme disease. The list of interventions was developed based on literature reviews concerning PILD recommended in the scientific and grey literature and was reviewed by a group of Lyme disease experts.

The PILD index is composed of four dimensions corresponding respectively to the following four specific groups of interventions that municipal authorities could implement: (PILD-1) Seeking information (e.g., whether the municipality is located in an area where people are at risk for Lyme disease), (PILD-2) discussing and implementing interventions (e.g., removing vegetation along walking paths), (PILD-3) informing the population (e.g., tips to remove the tick), (PILD-4) upstream actions (e.g., drafting a briefing note regarding Lyme disease to a decision-making (e.g., City Council) or advisory (e.g., Planning Advisory Committee) body). The interventions composing each of these four dimensions, the questions used in the survey, the response scales, and the method used to create the score are presented in Table 3.

#### 2.2.4. Creation of the PILD Index

The purpose of the first part of our analysis was to create the PILD index. To that end, item analysis and confirmatory factor analysis were performed. Because the creation of this index goes beyond the scope of this article, we will only briefly present the method used. 

Second, to determine which groups of interventions could be retained in the created index, we assessed its psychometric qualities. To that end, we performed an item analysis, using Samejima’s graded response model [60]. The objective of this item analysis was to assess the performance of the items according to certain psychometric parameters (e.g., the ability to distinguish between municipalities who implement PILD and those that do not) and to determine which items to retain in the final measure. This analysis led us to exclude interventions related to lawn mowing and grounds maintenance from the PILD index. Unlike those in the other four groups, these two interventions are not specific to municipalities who are more likely to implement preventive interventions for Lyme disease.

Third, we conducted a confirmatory factor analysis (CFA) to assess the unidimensionality of the index. We tested a parsimonious model that included the four retained interventions within a single construct representing the implementation of PILD. We then assessed the compatibility of the empirical data with this hypothetical measurement model using various fit indices as operationalized in Mplus 8 [61]. 

Finally, with the retained groups of interventions, we calculated a score of the municipalities’ levels of implementation of PILD. More specifically, for each respondent, we calculated the average score associated with the final four groups of interventions retained. This procedure was chosen to give the same weight to all groups of interventions.

### 2.3. Statistical Analysis

#### 2.3.1. Determinants of Municipal Authorities’ Implementation of PILD

For the purpose of identifying the factors leading municipal authorities to implement PILD and of determining whether perceived barriers moderate the intention-implementation relationship, we performed two statistical analyses. First, we tested a model that included TPB and HBM constructs in which the intention-implementation relationship was not moderated by the perceived barriers. Second, the model tested included a moderating effect of the perceived barriers on the intention-implementation relationship. 

Structural equation methods (SEM) with Mplus 8.0 [61] were used for all the analyses. It is recommended that multiple indicators be used for each latent variable in SEM because scores from multiple indicators tend to be more reliable and valid than those from a single indicator [62]. 

For the first model, weighted least square mean and variance adjusted estimation (WLSMV) was used because this estimator is more robust with categorical variables and large numbers of factors. The WLSMV estimation method also performs better than the MLR estimator in terms of computation time [63]. Because the second model included the test of a moderating effect, maximum likelihood estimation (ML) or maximum likelihood estimation with robust standard errors (MLR) were the estimators we could use. We chose MLR because this method is robust with respect to the non-normal distribution of scores [64].

Given the known oversensitivity of the chi-square test to sample size, minor deviations from normality, and minor model misspecifications, model fit is usually assessed with sample-size-independent fit indices, such as the comparative fit index (CFI), the Tucker-Lewis index (TLI), and the root mean squared error of approximation (RMSEA). According to conventional rules of thumb [62,65], acceptable and excellent model fit is indicated by CFI and TLI values greater than 0.90 and 0.95, respectively, and by RMSEA values smaller than 0.08 and 0.06, respectively.

Due to missing data, before performing SEM analyses, we ran an imputation method called MICE (Multiple imputation by chained equation) using the MICE package (version 3.3.0) for R [66]. For this analysis we used R (version 3.5.1) with RStudio (version 1.1.456), on PC [67]. We used sociodemographic variables, the TBP variables, and HBM variables for the imputation. Through this method, we generated 10 imputed datasets, which were then used for all the following analyses. The Mplus program was used to pool the results from the 10 datasets.

## 3. Results

### 3.1. Descriptive Statistics

Means (M) and standard deviations (SD) for each measure are reported in Table 4. Municipal officers reported that their municipality implemented a low number of PILD (M = 5.61 on a maximum score of 19 (theoretical range from 0 to 19), SD = 5.25). The mean intervention implementation score was 1.63/3 (SD = 1.31) for the group on seeking information, 2.55/9 (SD = 3.33) for the group on disseminating information to the population, 0.47/2 (SD = 0.71) for the group on interventions discussed and implemented, and 0.73/5 (SD = 1.04) for the group on upstream actions. 

We assessed the link between the municipalities’ levels of involvement in PILD and their location (i.e., the health regions), and their size. A two-way ANOVA was conducted to compare the level of PILD implementation between health regions and population sizes. There was at least one significant mean difference in the implementation of PILD between health regions: The *p* < 0.01 level (F(9,190) = 11.13, *p* < 0.0001). However, the mean score of PILD implementation did not differ according to the municipalities’ population size.

We then performed least squares means multiple comparisons using a Tukey-Kramer correction for health regions. The test indicated that the implementation of PILD was significantly higher in two of the 10 health regions: Monteregie and Estrie showed higher rates of implementation of PILD than eight and six of the other health regions, respectively. These two regions had the highest number by far of cases of Lyme disease in 2018 [11].

Regarding the TPB variables, municipal officers reported a moderate intention to implement PILD (M = 2.42 on a maximum score of 4, SD = 0.73), a strong attitude toward PILD (M = 2.71 on a maximum score of 4, SD = 0.61), moderate perceived social pressure (M = 2.23 on a maximum score of 4, SD = 0.75), and strong perceived barriers to (or weak perceived control over) the implementation of PILD (M = 3.06 on a maximum score of 4, SD = 0.47). Regarding perceived vulnerability to Lyme disease, municipal officers thought that the population was moderately at risk of contracting Lyme disease (M = 2.52 on a maximum score of 5, SD = 1.34). Furthermore, municipal officers perceived a strong risk that Lyme disease would have a negative impact on people’s health (M = 3.22 on a maximum score of 4, SD = 0.54). Results of correlations revealed that almost all the TPB variables correlated significantly with intentions or implementation of PILD, while HBM variables correlated significantly only with the implementation of PILD (see Table 4). 

### 3.2. Test of the TPB Model with HBM Constructs without Moderating Effect

**Figure 2:**
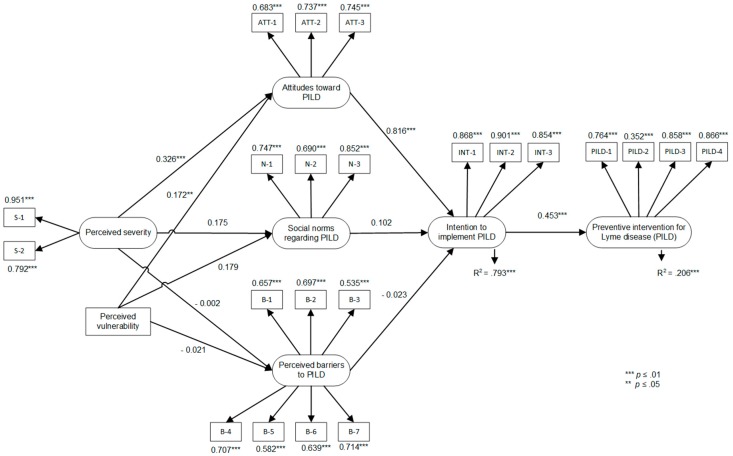
TPB and health belief model (HBM) variables predicting the implementation of preventive interventions for Lyme disease. *Note*. Correlations between predictor variables were: 0.584 *** between attitude and perceived social norms; −0.288 *** between attitudes and perceived control (barriers) over the implementation of PILD; −0.043 between social norms and perceived control (barriers) over the implementation of PILD; and 0.272 *** between perceived vulnerability and perceived severity. ** *p* < 0.05. *** *p* < 0.01.

The test of the TPB model showed that the model accounted for 79.3% of the variance in intentions to implement PILD and 20.6% of the variance in PILD implementation (see Figure 2). Among the proximal determinants of the TPB, only attitudes were significantly associated with intentions (standardized beta, β = 0.816, *p* < 0.01). The association of intentions (β = 0.453, *p* < 0.01) with the implementation of PILD was significant. The fit of the model to the data was excellent: CFI = 0.932, TLI = 0.921 and RMSEA = 0.074, χ^2^(217) = 419.89. 

### 3.3. Test of the TPB Model with HBM Constructs with Moderating Effect

**Figure 3:**
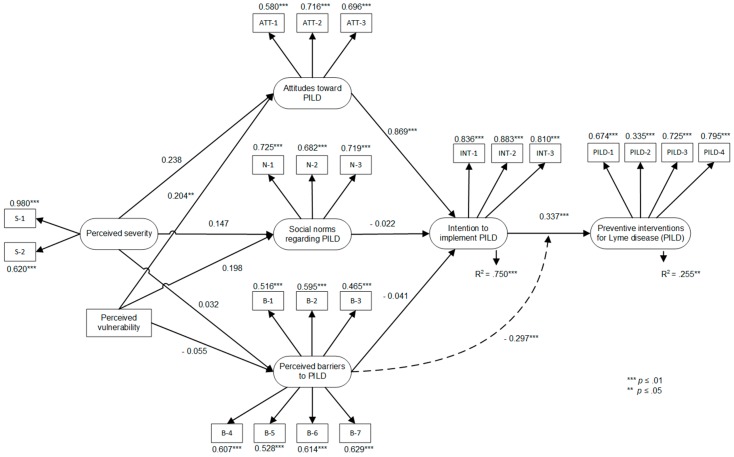
TPB and HBM variables predicting the implementation of preventive interventions for Lyme disease, with moderation effect. *Note*. Correlations between predictor variables were: 0.590 *** between attitude and perceived social norms; −0.192 between attitudes and perceived control (barriers) over the implementation of PILD; −0.041 between social norms and perceived control (barriers) over the implementation of PILD; 0.256 ** between perceived vulnerability and perceived severity. ** *p* < 0.05. *** *p* < 0.01.

In the second analysis, we tested the same model, with the exception that a moderating effect on the intention-implementation relationship was added in the former model (Figure 3). No model fit information is given when a model includes an interaction term. The percentage of variance explained by this second model was higher for the prediction of the implementation of PILD (25.5%, that is + 4.9%), but lower for the prediction of the intentions to implement PILD (75.0%, that is −4.3%). Notably, the model showed that the intention-PILD implementation association was moderated by the perceived control (barriers) over the implementation of PILD (β = −0.297, *p* < 0.01). This means that the strength of the association between intentions and the implementation of PILD was reduced when municipal officers perceived barriers to implementing PILD. 

Both models showed that attitudes were significant predictors of intentions to implement PILD. Moreover, the association between municipal officers’ attitudes toward the adoption of PILD and their behavioral intentions was stronger in the model that included the moderating effect (β = 0.869 comparatively to 0.816). 

## 4. Discussion

### 4.1. Implementation of Preventive Interventions for Lyme Disease by Municipal Authorities

The creation of a validated, rigorous index formed from a set of indicators referring to interventions that municipal officers could implement to prevent Lyme disease allowed us to draw a portrait of the actions undertaken regarding this issue by the 820 Quebec municipalities that are at risk for Lyme disease. Thanks to this index, it will be possible to evaluate the evolution over time of their adaptation to Lyme disease.

The portrait of the preventive interventions for Lyme disease implemented by the 820 Quebec municipalities indicates that municipal authorities are moderately active in seeking information on whether their municipality is in an area where people are at risk for Lyme disease, about ways to better prevent Lyme disease, or regarding the impact that Lyme disease may have on physical or mental health. This result suggests that some municipal authorities are aware of the Lyme disease situation and are indeed eager to know more about it, but that many municipalities are still lagging on that matter.

Making information available to the population was the second most common group of actions among the interventions evaluated. This is not surprising, as according to Piesman and Eisen [27], this category of measures, which refers to education and sensitization of at-risk populations, is currently the main public health strategy adopted by most countries. In this regard, various activities that engage communities and workers, which are not education/sensitization actions, are starting to take off in Quebec [68]. 

These results are also similar to those from other studies on tracking the progress of adaptation, which indicate that adaptations being implemented by municipalities and civil society organizations in Canada consist predominantly of groundwork interventions, such as awareness, research, and networking activities that aim to build adaptive capacity [24,54,69]. Still, municipalities’ adaptation to Lyme disease does not involve many specific adaptation actions, not even the development and planning stages to implement such actions. In fact, upstream actions like drafting briefing notes, proposing actions, working on developing partnerships with other organizations, and working on action plans, were seldom mentioned by respondents (see Section 3.1). 

Our results also show that municipalities rarely implement preventive environmental measures aimed at protecting the population from ticks. We have not identified any similar studies at the organizational level, but those at the individual level have shown that individual homeowners or homeowner associations tend to rely on public service interventions [70]. This may suggest that a reverse phenomenon would be at play at the municipal level. Because tick control could be seen as an individual responsibility, efforts by the municipality would be limited mainly to seeking information and communicating it to the population, with the idea of encouraging the population to adopt preventive behaviors. This specific barrier could be interesting to investigate in future research in a municipal setting. As indicated earlier in the introduction, municipalities in the province of Quebec do not have an official mandate at this time to act on Lyme disease. The responsibility for public health interventions rests with the Ministry of Health and Social Services. The option for municipalities to be more active in the prevention of Lyme disease is still being debated. There may thus be a gap, given that the risk for this disease varies substantially within small geographical areas. Preventive interventions that are most likely to be effective should be implemented at the local level. In this regard, municipalities have an advantage, which, to date, has not been exploited. This is different from other Canadian provinces, where public health officers are always visible at the municipal level.

### 4.2. Factors Leading Some Municipalities to Be Active in Lyme Disease Prevention

Overall, the results of this study showed the usefulness of combining TPB and HBM as a framework for understanding the implementation of preventive interventions for Lyme disease, as well as the intentions to implement such interventions by municipal officers. Our models demonstrated good fit, and the introduction of a moderating effect of the perceived barriers in the intention-implementation relationship improved by 4.9% the level of explained variance in intervention implementation. Finally, our questionnaire explained a high percentage of the intention variance (75%), making it a good questionnaire for further studies in this field.

Our results partially confirmed our first and second hypotheses: Whereas only one proximal factor of the TPB (attitudes) was significantly associated with behavioral intentions, behavioral intentions were a significant predictor of the adoption of preventive behaviors. However, the perception of the social norms regarding PILD was not a significant predictor of the intentions to implement PILD. This means that such intentions and actions are less the result of pressure felt by municipal officers from regional bodies, public health authorities, and the population, for example, than of municipal officers’ positive attitudes toward the importance of their municipality’s actions regarding Lyme disease. This result is consistent with findings from a similar study, targeting municipal officers in the province of Quebec but pertaining to the adoption of heat and flood adaptation behaviors [54]. Indeed, that study also showed that social norms were not predictors of intentions to act. 

The model did not show a significant relationship between perceived control (barriers) over the implementation of PILD and intention to implement PILD. Instead, our results indicated that perceived barriers to PILD played a significant role in the model, but by negatively moderating the strength of the relationship between intentions and actual implementation of PILD, which is in line with our third hypothesis. As expected, respondents showed a high level of perceived barriers (score of 3.06/4, the highest for a proximal factor). This is an interesting finding, given that relatively few studies have submitted this hypothesis to empirical testing. When it was empirically tested in other studies, the interaction term was often not significant, and even when it did have a significant regression coefficient, it tended to account for little or no additional variance in the prediction of behaviors; for a review on this issue, see Fishbein and Ajzen [52]. In our case, the addition of the moderating effect increased the proportion of explained variance in intervention implementation by 4.9%. However, this increase came at the cost of a decrease in the proportion of explained variance in intentions (decrease of 4.3%). This result, however, must be interpreted with caution, as it is impossible to know whether this increase in the variance explained is due simply to the addition of the interaction term or to the change of the estimation method (MLR instead of WLSMV).

This could indicate that whereas showing a strong predisposition on the respondent’s part to act on the prevention of Lyme disease, this intention comes up against a certain reality of barriers that impede the authorities’ actual capacity to act. This is an important finding, as urban planners or environmental officers may be unable to truly act on their intentions despite displaying a positive attitude toward the implementation of PILD. A closer look at the specific barriers shows that budget (B-7), difficulties achieving joint action with other regional actors (B-6), and workload and overburden (B-4) appear to be the most important barriers and are all factors over which municipal officers have little or no control. 

Finally, in line with our fourth hypothesis, perceived severity of the impacts of Lyme disease on physical and mental health and perceived vulnerability (i.e., whether respondents feel that it is possible to contract Lyme disease in their municipality) appear to be linked to attitudes toward PILD. However, the model without moderating effect indicated a small but statistically significant effect size (β = 0.326, *p* < 0.01) of perceived severity on attitudes toward PILD, while in the model with moderating effect, the effect size was smaller and non-significant (β = 0.238, *p* = 0.101). Still, this result is worthwhile as an effect size above 0.2 is generally considered as “practically” significant [71]. This means that municipal officers who perceive high severity and vulnerability tend to show a more positive attitude toward the intention to implement PILD. Such background factors provide valuable information about the elements to target in the context of training modules for municipal staff, with a view to strengthening municipal officers’ attitudes, and then improving their intentions to implement PILD.

This study has some limitations, such as its reliance on self-reports of PILD implementation and the possibility of respondents overestimating the extent to which their municipality implemented these interventions. Of course, it would have been virtually impossible to obtain objective measures for the wide variety of interventions we tried to assess and, in any case, the present study is comparable in this regard to most other studies on pro-environmental behavior [72,73,74]. Our assurance to the participants that their responses were anonymous was designed to mitigate the tendency toward socially desirable responding.

Although consistent with findings in other behavioral domains, a second limitation is that the TPB model (to which we added two variables from the HBM) predicted only 25.5% of our general measure of PILD implementation. This may be in part because we were not trying to predict the implementation of one specific intervention but rather a series of interventions, which is much harder to accomplish. As Fishbein and Ajzen [52] stipulate, in order to obtain an accurate prediction, the context in which the behavior/intervention takes place needs to be specific. In our case, municipalities were our level of analysis, while our respondents were municipal officers that may not have known all the actions that their municipalities had adopted to prevent Lyme disease. Furthermore, the person who responded to our questionnaire may not be the same person who acted, which means that the target element of the intention differed from the target element of the behavior/intervention. This situation, described by Fishbein and Ajzen [52] as behavioral incompatibility, could account for the large difference in explained variance between intention (75.0%) and PILD implementation (25.5%).

A third limitation relates to the validity of our PILD implementation measure. Although we consulted people working in municipalities, as well as public health experts on Lyme disease, our protocol did not include a pilot qualitative study. This would have provided a deeper understanding of municipal authorities’ behavioral beliefs, social norm beliefs, and control beliefs, as well as other examples of possible PILD. Such a study might help to refine, update, and adapt our tool and questionnaire to make them fit the context of municipal officers who are responsible for implementing PILD. Conducting focus groups or interviews with municipal representatives would have helped produce a more accurate measurement of the subjective dimensions in our model (beliefs, attitudes, perceptions, etc.). That said, since our goal was to develop an index, the research design could not be based solely on qualitative methods, since it would be impossible to generate enough observations to validate the psychometric quality of the measurement instrument. To ensure the validity of the PILD index, a future study should be conducted to reproduce the index and verify its invariance in time and its factor stability. Focus groups could then be used to identify the beliefs underlying attitudes and perceptions among municipal officials. 

As a fourth limitation, although our study had an interesting response rate (36%), we cannot rule out the risk of selection bias and a response bias, where the respondents may be different from the non-respondents and may not be entirely representative of our study population. Furthermore, in order to keep the questionnaire at a reasonable length (and to maximize our chances of obtaining a good response rate), we had to give up the measurement of certain variables. For this reason, we did not measure behavioral, injunctive norm, or control beliefs, which would have enhanced our understanding of specific beliefs and their influence on the proximal antecedents of intentions, that is, attitudes, subjective norms, and perceptions of behavioral control. 

Finally, our database had variables with high levels of missing values. For this reason, we were limited in the number of observations and of parameters we could include in our models. For instance, we were also unable to include the “perceived effectiveness of preventive intervention” variable in our model because many respondents said they could not answer such a question, not having tried the measures mentioned themselves. 

## 5. Conclusions

In conclusion, our results suggest that whereas working on attitudes is important, it will not be sufficient to overcome the real (or perceived) barriers. Given that municipal officers have little control over such barriers as non-availability of a special budget or the absence of prioritization by elected officials, training content should aim specifically at reinforcing their skills to enable them to integrate Lyme disease prevention in actions or decision-making processes that they can actually influence. As it is the case with adaptation to climate change in general, Lyme disease prevention initiatives are likely to be mainstreamed, in other words, to be integrated into existing projects, actions, plans, strategies, etc. [75]. Given municipal staff’s lack of control, the mainstreaming of Lyme disease prevention appears to be a promising strategy, provided that they develop the skills needed to incrementally integrate Lyme disease prevention at their level of action [76].

## Figures and Tables

**Table 1:** Response rate by health region.

Quebec Health Region	Response Rate by Region (n/N) ^a^	Percentage of Reported Human Cases of Lyme Disease (Acquired Locally) in 2018
Capitale-Nationale	25/59 (42%)	0 (0%)
Mauricie-Centre du Québec	34/121 (28%)	17 (8%)
Estrie	28/89 (31%)	87 (40%)
Montreal	12/34 (35%)	1 (0.5%)
Outaouais	27/67 (40%)	2 (0.9%)
Chaudière-Appalaches	37/136 (27%)	0 (0%)
Laval	0/1 (0%)	0 (0%)
Lanaudière	25/57 (44%)	2 (0.9%)
Laurentides	34/76 (45%)	2 (0.9%)
Montérégie	71/177 (40%)	93 (42%)
Other ^b^		15 (6.8%)
TOTAL	293/817 (36%)	219 (100%)

^a^ n = number of municipalities who answered the survey, N = number of municipalities in each health region, ^b^ Official data available for 2018 show 14 cases for which it was not possible to identify the region of acquisition. Another case was acquired in a health region (Côte-Nord) that, when the survey was administered, was not identified as a region with a higher risk for Lyme disease.

**Table 2:** Sample size by municipality size.

Municipality Sizes	Number	%
1–499	42	14%
500–999	67	23%
1000–1999	44	15%
2000–2999	31	11%
3000–3999	16	6%
4000–4999	10	3%
5000–9999	35	12%
10,000–49,999	30	10%
50,000 +	18	6%
TOTAL	293/820	100%

**Table 3:** Implementation of preventive interventions for Lyme disease measured at the municipal authorities’ level (PILD index).

Questions Used and Preventive Interventions		Scale	Method Used to Create the Score (Min. and Max.)
Seeking information (PILD-1)	Using the following scale of responses, please indicate if you, or someone else employed by your municipality, has already inquired about... Whether the municipality is located in an area where people are at risk for Lyme disease About ways to better prevent Lyme disease About the impact Lyme disease may have on physical or mental health	(1) Yes, (0) No	Sum of all 3 items (Min = 0; Max = 3)
Actions discussed and actions implemented (PILD-2)	Using the following scale, please indicate the extent to which staff in your municipality have, over the last 2 years, (discussed/implemented) the following actions to protect the population from Lyme disease. Separate woodlots from the lawn with wood chips, mulch, or gravel Place children’s games and sandboxes away from the edge of woodland Place children’s games and sandboxes that are near woodlots on a structure made of woodchips or mulch Protect areas surrounding buildings located near wooded areas by pavement, low wall, or plantation container Remove vegetation along walking paths Delineate public access woodlots with gates to keep away deer	(0) Never, (1) To my knowledge this was discussed at an official meeting, (2) This has been implemented	Sum of all 6 items (Min = 0; Max = 12) (6–12) A majority of the actions were implemented. Final score of 2. (1–5) Few actions were implemented or discussed. Final score of 1. (0) Nothing was discussed or implemented. Final score of 0. (Min = 0; Max = 2)
Information to the population (PILD-3)	Using the following scale of response, please indicate whether your municipality has already made available information about Lyme disease, for example on the municipality’s website, via flyers, or information boards at the parks entrance. List of nine types of information made available about Lyme disease, (e.g., on the municipality’s website, in leaflets, or on information boards at park entrances) Areas where people are at risk of acquiring Lyme disease in the area A description of the disease A description of the tick A description of the symptoms Possible protection and prevention measures Tips to remove the tick Information on what to do with the tick once removed References for more information on the disease References about who and when to consult for medical advice	(1) Yes, (0) No	Sum of all 9 items Min = 0; Max = 9
Upstream actions (PILD-4)	Have you, or anyone else employed by your municipality, ever… Read the information provided by the staff of a regional health and social services center about the municipality being located on a territory where people are at risk of contracting Lyme disease Drafted a briefing note regarding Lyme disease to a decision-making (e.g., City Council) or advisory (e.g., Planning Advisory Committee) body Proposed action recommendations for Lyme disease to a decision-making (e.g., City Council) or advisory (e.g., Planning Advisory Committee) body Worked to develop partnerships or collaborations with other organizations (e.g., a health and social services center, department of public health, health and social services ministry) with the aim of dealing with Lyme disease Worked to develop an action plan for the municipality with respect to Lyme disease	(1) Yes, (0) No	Sum of all 5 items Min = 0; Max = 5

**Table 4:** Means, standard deviations, and correlations between the theory of planned behavior (TPB) variables, perceived vulnerability, and perceived severity.

Variables	# of Items	*M*	SD	1	2	3	4	5	6	7
1. PILD index	4	5.61	5.25	-						
2. Intention	3	2.42	0.73	0.322 **	-					
3. Attitude	3	2.71	0.61	0.236 **	0.683 **	-				
4. Perceived social norms	3	2.23	0.75	0.305 **	0.415 **	0.481 **	-			
5. Perceived control (barriers)	7	3.06	0.47	−0.042	−0.242 *	−0.211	-0.028	-		
6. Perceived vulnerability	1	2.52	1.34	0.479 **	0.131	0.131	0.075	0.059	-	
7. Perceived severity	2	3.22	0.54	0.218 **	0.157	0.213*	0.161	−0.029	0.230 **	-

Notes. Theoretical range for the PILD index: [0,19]; Theoretical range for intention: [1,4]; Theoretical range for attitude: [1,4]; Theoretical range for social norms: [1,4]; Theoretical range for perceived control (barriers): [1,4]; Theoretical range for perceived vulnerability: [0,5]; Theoretical range for perceived severity: [1,4]. * *p* < 0.05. ** *p* < 0.01.
